# Primary Oral Health Care in Reducing Prosthodontic Burden: A Review

**DOI:** 10.7759/cureus.106122

**Published:** 2026-03-30

**Authors:** Sumeet Agarwal, Laresh N Mistry, Barun Kumar, Saloni Mistry, Arshia Baig, Sayem A Mulla

**Affiliations:** 1 Prosthodontics, Bharati Vidyapeeth (Deemed to be University) Dental College and Hospital, Navi Mumbai, IND; 2 Pedodontics and Preventive Dentistry, Bharati Vidyapeeth (Deemed to be University) Dental College and Hospital, Navi Mumbai, IND; 3 Oral and Maxillofacial Surgery, Bharati Vidyapeeth (Deemed to be University) Dental College and Hospital, Navi Mumbai, IND; 4 Prosthodontics, Dr. GD Pol Foundation YMT Dental College and Hospital, Navi Mumbai, IND; 5 Conservative Dentistry and Endodontics, Sharad Pawar Dental College and Hospital, Wardha, IND; 6 Dentistry, Bharati Vidyapeeth (Deemed to be University) Dental College and Hospital, Navi Mumbai, IND

**Keywords:** oral health, primary oral health, prosthodontic, public health, public health awareness

## Abstract

The cumulative effect of untreated oral illnesses, tooth loss, and functional degradation over time is represented by prosthodontic treatment demands. Due to aging populations, high rates of periodontal disease and tooth caries, limited access to early dental treatment, and socioeconomic inequities, prosthodontic care is becoming more and more necessary in many communities, especially in low- and middle-income nations. In order to lessen this prosthodontic load, Primary Oral Health Care (POHC), which is based on the ideas of prevention, early intervention, equality, and community involvement, is essential. In this narrative review, the conceptual framework of POHC is explored, the etiological pathways leading to prosthodontic need are examined, and the effectiveness of primary oral health strategies in reducing tooth loss, delaying prosthetic rehabilitation, and improving oral health-related quality of life (OHRQoL) is critically examined. In order to lessen the long-term prosthodontic load, the analysis emphasizes policy implications, workforce issues, and future objectives for incorporating POHC into complete oral health systems.

## Introduction and background

Prosthodontic burden, which is the population-level need for tooth replacement and the restoration of oral function and aesthetics, is a significant global public health issue [1]. Tooth loss is still a major oral health concern globally, despite tremendous progress in restorative and preventative dentistry. It is mostly caused by avoidable diseases that disproportionately impact disadvantaged and underprivileged people, such as dental caries, periodontal disease, trauma, and oral infections. The prosthodontic load is made worse by socioeconomic inequities, poor oral health awareness, aging populations, and restricted access to dental treatment, especially in low- and middle-income nations. The cumulative consequences of untreated oral disorders raise the requirement for extensive prosthodontic rehabilitation as life expectancy rises worldwide, further taxing healthcare systems [1].

Tooth loss has far-reaching effects on general health and well-being in addition to functional limitations such as impaired speech and mastication. It negatively impacts nutrition by restricting dietary choices, frequently resulting in diets that are low in important nutrients, hence increasing the risk of systemic illnesses such as cardiovascular disease, diabetes, and frailty in older persons. Missing teeth can also have negative visual and social effects on one's self-worth, social interactions, and psychological well-being, which can lead to social disengagement, anxiety, and despair. When taken as a whole, these functional, psychosocial, and systemic effects highlight how crucial it is to address prosthodontic burden through integrated public health strategies that emphasize early intervention, prevention, and fair access to oral healthcare services in addition to rehabilitative care [2].

Prosthodontic treatment has always been seen as a tertiary-level intervention that prioritizes rehabilitation above prevention. However, a cycle of illness, tooth loss, and repetitive prosthetic replacement is sustained by an overdependence on prosthetic solutions without addressing upstream causes [3]. In line with the World Health Organization's more general Primary Health Care (PHC) strategy, Primary Oral Health Care (POHC) places a strong emphasis on community-based health promotion, early diagnosis, and disease prevention. POHC has the ability to dramatically lower the frequency and severity of problems that lead to prosthodontic demands by acting early in the illness continuum [4].

The purpose of this narrative review is to summarize the available data about POHC's contribution to prosthodontic burden reduction and to emphasize its significance in contemporary prosthodontic practice and oral health policy.

## Review

Methodology

To conduct this narrative review, scientific databases including PubMed, Scopus, Web of Science, and Google Scholar were systematically searched for English-language literature up to 2025. Among the terms used were prosthodontic burden and basic oral healthcare. Additional items were discovered by cross-referencing relevant bibliographies. All kinds of publications were included in order to have a comprehensive understanding of the relationship. After screening, a total of 23 articles were included (Table 1).

**Table 1:** Summary of the included studies UHC: universal health coverage, LMIC: low- and middle-income country, POHC: Primary Oral Health Care, PHC: Primary Health Care, OHRQoL: oral health-related quality of life Table credit: Dr. Sayem Mulla and Dr. Laresh Mistry

Author(s) and year	Study focus	Key findings/take-home messages
Ardakani and Bayati (2025) [1]	Oral health coverage and UHC	Oral health remains poorly integrated into UHC globally; preventive and primary oral care services are inconsistently financed and delivered, especially in LMICs.
Hyde et al. (2017) [2]	Prevention of tooth loss and pain	Early prevention of caries and periodontal disease significantly reduces tooth loss, dental pain, and long-term prosthodontic demand.
Aljulayfi (2024) [3]	Prosthodontic management of tooth wear	Emphasizes conservative and preventive strategies before extensive prosthodontic rehabilitation to reduce biological and economic burden.
Honkala (2014) [4]	POHC	POHC is foundational for prevention, early diagnosis, and reduction of advanced oral disease requiring complex prosthodontic care.
Prasad et al. (2019) [5]	Integration of oral health into PHC	Strong evidence supports integrating oral health into primary health systems to improve access, reduce inequalities, and lower treatment burden.
Bourgeois et al. (2014) [6]	Oral disease prevention in PHC	Prevention-based oral health education within PHC is cost-effective and essential for reducing future restorative and prosthetic needs.
Ali et al. (2019) [7]	OHRQoL after prosthodontic treatment	Prosthodontic rehabilitation improves OHRQoL, but does not fully compensate for delayed or absent preventive care.
de Lucena et al. (2021) [8]	Socioeconomic determinants	Lower socioeconomic status is strongly associated with higher oral disease burden and increased prosthodontic treatment needs.
Gilbert et al. (2003) [9]	Social determinants of tooth loss	Tooth loss is driven more by social, behavioral, and access-related factors than purely clinical causes.
Al-Harbi and El Tantawi (2017) [10]	Normative prosthodontic needs	High prosthodontic needs negatively impact daily life and psychosocial well-being, even among younger populations.
Kashbour et al. (2020) [11]	Sealants versus fluoride varnish	Preventive interventions such as sealants and fluoride varnishes are effective in reducing caries and future prosthetic requirements.
Frencken et al. (2012) [12]	Minimal intervention dentistry	Minimal intervention dentistry reduces disease progression and delays the need for extensive prosthodontic rehabilitation.
Yeung et al. (2020) [13]	Immediate complete dentures	Timely prosthodontic rehabilitation improves function and adaptation but requires follow-up and preventive maintenance.
Meyer et al. (2008) [14]	Tooth loss and systemic disease	Tooth loss and periodontal disease are linked to systemic conditions, highlighting broader health implications of oral neglect.
Matthews (2014) [15]	Periodontal care in primary care	Periodontal disease prevention and early management in primary care reduce long-term tooth loss and prosthetic demand.
Nikolovska et al. (2018) [16]	Prosthodontic needs in the elderly	Elderly populations show high unmet prosthodontic needs due to access, affordability, and service availability barriers.
Shen et al. (2025) [17]	Domiciliary denture care	Home-based prosthodontic services improve access and oral function in dependent and institutionalized older adults.
Sharma and Singh (2024) [18]	Prosthodontic status in India	High prevalence of unmet prosthodontic needs reflects gaps in preventive care and early intervention at the primary level.
Probst et al. (2019) [19]	Cost-effectiveness of implants	Implant-supported prostheses may be cost-effective long term, but remain inaccessible for many populations without public funding.
Lima de Paula et al. (2019) [20]	Psychosocial impact of tooth loss	Socioeconomic status influences emotional response, acceptance, and success of prosthodontic rehabilitation.
Sikdar et al. (2025) [21]	Prosthodontics in palliative care	Prosthodontic care plays a critical role in comfort, dignity, and quality of life in palliative settings.
Hung et al. (2025) [22]	Interprofessional oral care	Integrating dentistry into interprofessional healthcare improves patient outcomes and reduces fragmented, late-stage care.
Patrick et al. (2006) [23]	Oral health disparities	Social and cultural determinants drive oral health inequities, reinforcing the need for community-based preventive strategies.

Conceptual framework of Primary Oral Health Care

The PHC paradigm is expanded upon by Primary Oral Health Care, which incorporates oral health into general health systems. Accessibility, affordability, community involvement, intersectoral collaboration, and a focus on preventative care are among its fundamental tenets. Instead of then replacing missing teeth, POHC emphasizes preserving the natural dentition throughout life [5]. POHC's essential elements include promotion and education of oral health, use of fluoride and dietary guidance to prevent oral disorders, early identification and treatment of periodontal disease and tooth caries, risk evaluation, customized preventative measures, and systems of referral for specialist treatment [6].

POHC stops the development of oral disorders before they reach advanced and irreversible stages by bolstering these preventative components. Timely, less invasive treatments are made possible by early detection and treatment of periodontal disease and caries. This method lessens the possibility of extractions by preserving natural teeth and supporting tissues. As a result, there is far less need for complicated and expensive prosthodontic therapy [7].

Prosthodontic burden: Etiology and determinants

A complex interplay of biological, behavioral, socioeconomic, and health system variables results in the prosthodontic burden. Globally, periodontal disease and dental caries continue to be the primary causes of tooth loss. These disorders are largely caused by poor oral hygiene habits, excessive sugar intake, tobacco use, and insufficient usage of dental services [8].

Disadvantaged communities have greater rates of untreated oral illness and quicker tooth loss due to socioeconomic inequities. Tooth extraction is the most popular and reasonably priced alternative since delayed treatment is frequently caused by limited access to dental care, inadequate oral health knowledge, and budgetary limitations. This eventually results in partial or whole edentulism, which raises the need for permanent or removable prostheses [9].

Importantly, prosthodontic need is not just a single clinical condition; rather, it represents the cumulative failure of prompt primary and preventive oral health treatment. Minor oral disorders such as incipient caries, plaque, and calculus can develop into tooth loss and functional impairment due to inadequate access to early diagnosis, preventative measures, and health education. This trend is made worse by inadequate continuity of care, restricted service coverage, and socioeconomic inequality. As a result, systemic inadequacies in oral healthcare delivery systems are indicated downstream by the demand for prosthodontic rehabilitation [10].

Role of POHC in preventing tooth loss

Preventing tooth loss, which is the main cause of prosthodontic burden, is one of POHC's most important contributions. It has been demonstrated that preventive measures such as topical fluoride treatment, pit and fissure sealants, community water fluoridation, and school-based oral health initiatives greatly lower the incidence of caries [11].

Preventive resin restorations and non-surgical periodontal treatment are examples of minimally invasive procedures that are made possible by early diagnosis of carious lesions and periodontal disease through regular screenings. By maintaining periodontal support and tooth structure, these actions extend the useful life of natural teeth [12].

By reducing the frequency and severity of oral disorders, Preventive Oral Health Care (POHC) immediately lowers the need for tooth extractions and subsequent prosthodontic rehabilitation. Through early identification and appropriate care of dental caries and periodontal problems, POHC helps retain natural dentition and maintain oral function. Frequent preventative measures, such as risk-based monitoring, fluoride administration, professional cleaning, and oral health education, slow the course of illness and its effects. By limiting tooth loss, POHC decreases the long-term clinical, economic, and emotional burden associated with prosthodontic therapy. Consequently, it promotes sustainable oral healthcare systems and enhances overall quality of life at the community level [13].

POHC and periodontal health preservation

One of the main causes of adult tooth loss and, hence, the need for prosthodontics is periodontal disease. As essential elements of oral health treatment, POHC places a strong emphasis on plaque control, tobacco cessation counseling, and routine periodontal evaluations [14].

Timely intervention is facilitated by training auxiliary staff and primary care dentists to recognize early indicators of periodontal disease. By stopping the course of the disease and preserving periodontal stability, non-surgical periodontal treatment administered at the primary care level might lessen the need for intricate prosthodontic solutions such as splinting, overdentures, or implant-supported prostheses [15].

Impact of POHC in geriatric and special populations

Because systemic comorbidities and cumulative oral disease load raise prosthodontic demands, aging people pose special concerns. Preventive treatment, upkeep of current restorations, and preservation of the remaining dentition are the key goals of POHC models designed for senior citizens [16].

Access constraints are addressed via domiciliary dental treatment, community-based outreach initiatives, and the incorporation of oral health within geriatric primary care services. POHC can postpone edentulism and lessen the need for full dentures or intricate implant therapy by emphasizing preventative and maintenance care [17].

Economic impact and health system benefits

Prosthodontic procedures demand a lot of resources, including specialized supplies, laboratory assistance, and qualified personnel. On the other hand, basic and preventive oral health therapies are scalable and reasonably priced [18].

By reducing the need for sophisticated prosthodontic treatments and frequent prosthesis replacement, investing in POHC lowers long-term costs [19]. From the standpoint of health systems, this change relieves the strain on tertiary care institutions and frees prosthodontists to concentrate on complicated rehabilitative situations instead of avoidable tooth loss [20].

Integration of prosthodontics with POHC

Preventive and maintenance-oriented care is becoming more and more important in modern prosthodontics. By promoting tooth-preserving techniques, creating prostheses that promote oral hygiene, and instructing patients on long-term care, prosthodontists play a crucial role in POHC [21].

Referral routes are strengthened and continuity of care is ensured by interdisciplinary collaboration between prosthodontists, public health dentists, and primary care physicians. Prosthodontic practice is in line with public health objectives of illness prevention and health promotion through such integration (Table 2) [22].

**Table 2:** Primary Oral Health Care strategies and their role in reducing prosthodontic burden POHC: Primary Oral Health Care Table credits: Dr. Sayem Mulla and Dr. Laresh Mistry

POHC component	Core interventions	Primary outcome	Effect on prosthodontic burden
Health promotion	Oral hygiene education, diet and tobacco counseling	Improved oral health behavior	Reduced disease progression and tooth loss
Preventive care	Fluoride use, sealants, community programs	Caries prevention	Lower incidence of partial edentulism
Early detection	Screening, risk assessment	Early disease control	Decreased need for extractions
Primary-level treatment	ART, basic restorations, non-surgical periodontal therapy	Tooth preservation	Reduced demand for prostheses
Geriatric and special care	Preventive maintenance, domiciliary care	Prolonged tooth survival	Delayed need for complete dentures

Challenges and future directions

Despite its advantages, there are obstacles to POHC implementation, including a lack of finance, unequal dental service distribution, a paucity of workers, and a lack of connection with general healthcare systems. Community involvement, curricular changes that prioritize preventative dentistry, and policy-level commitment are all necessary to overcome these obstacles [23].

Longitudinal evaluations of POHC therapies and their effects on prosthodontic outcomes should be the main focus of future studies. Evidence-based planning will be further informed by bolstering surveillance systems to monitor tooth loss and prosthodontic requirements.

## Conclusions

By avoiding oral illnesses, maintaining natural dentition, and addressing socioeconomic causes of tooth loss, Primary Oral Health Care plays a critical role in lowering the prosthodontic burden. POHC improves oral health outcomes for individuals, as well as the efficiency and equality of the health system, by reorienting the focus from rehabilitative to preventative treatment. For oral healthcare to be delivered sustainably and to reduce the long-term need for prosthetic rehabilitation, POHC concepts must be included in prosthodontic practice and policy.

## References

[1] Ardakani MS, Bayati M (2025). Global situation of oral health coverage toward universal health coverage: a scoping review. Prev Med Rep.

[2] Hyde S, Dupuis V, Mariri BP, Dartevelle S (2017). Prevention of tooth loss and dental pain for reducing the global burden of oral diseases. Int Dent J.

[3] Aljulayfi IS (2024). Management of prosthodontic challenges in tooth wear cases: a narrative review. J Pharm Bioallied Sci.

[4] Honkala E (2014). Primary oral health care. Med Princ Pract.

[5] Prasad M, Manjunath C, Murthy AK, Sampath A, Jaiswal S, Mohapatra A (2019). Integration of oral health into primary health care: a systematic review. J Family Med Prim Care.

[6] Bourgeois DM, Phantumvanit P, Llodra JC, Horn V, Carlile M, Eiselé JL (2014). Rationale for the prevention of oral diseases in primary health care: an international collaborative study in oral health education. Int Dent J.

[7] Ali Z, Baker SR, Shahrbaf S, Martin N, Vettore MV (2019). Oral health-related quality of life after prosthodontic treatment for patients with partial edentulism: a systematic review and meta-analysis. J Prosthet Dent.

[8] de Lucena EH, da Silva RO, Barbosa ML, de Araújo EC, Pereira AC, Cavalcanti YW (2021). Influence of socioeconomic status on oral disease burden: a population-based study. BMC Oral Health.

[9] Gilbert GH, Duncan RP, Shelton BJ (2003). Social determinants of tooth loss. Health Serv Res.

[10] Al-Harbi F, El Tantawi M (2017). Normative prosthodontic care need: does it impact the daily life of young Saudis with high level of oral diseases? A cross sectional study. BMC Oral Health.

[11] Kashbour W, Gupta P, Worthington HV, Boyers D (2020). Pit and fissure sealants versus fluoride varnishes for preventing dental decay in the permanent teeth of children and adolescents. Cochrane Database Syst Rev.

[12] Frencken JE, Peters MC, Manton DJ, Leal SC, Gordan VV, Eden E (2012). Minimal intervention dentistry for managing dental caries - a review: report of a FDI task group. Int Dent J.

[13] Yeung C, Leung KC, Yu OY, Lam WY, Wong AW, Chu CH (2020). Prosthodontic rehabilitation and follow-up using maxillary complete conventional immediate denture. Clin Cosmet Investig Dent.

[14] Meyer MS, Joshipura K, Giovannucci E, Michaud DS (2008). A review of the relationship between tooth loss, periodontal disease, and cancer. Cancer Causes Control.

[15] Matthews DC (2014). Prevention and treatment of periodontal diseases in primary care. Evid Based Dent.

[16] Nikolovska J, Korunoska-Stevkovska V, Mijoska A, Popovska L (2018). Prosthodontics status and treatment needs among the elderly in the Republic of Macedonia. Open Access Maced J Med Sci.

[17] Shen X, Chen X, Lei S, Cheng X, Chen Q, He F, Chen Z (2025). Domiciliary denture treatment for edentulous older adults: a retrospective study. BMC Oral Health.

[18] Sharma AK, Singh K (2024). Assessment of prosthodontic status and treatment needs of patients visiting dental institutions of Punjab. J Pharm Bioallied Sci.

[19] Probst LF, Vanni T, Cavalcante DF, Silva ET, Cavalcanti YW, Passeri LA, Pereira AC (2019). Cost-effectiveness of implant-supported dental prosthesis compared to conventional dental prosthesis. Rev Saude Publica.

[20] Lima de Paula LM, Sampaio AA, Costa JG, Gomes VE, Ferreira EF, Ferreira RC (2019). The course from tooth loss to successful rehabilitation with denture: feelings influenced by socioeconomic status. SAGE Open Med.

[21] Sikdar C, Srivastava SK, Rana A, Srivastava S, Shekhar A (2025). Prosthodontics in palliative care: optimizing oral rehabilitation for quality of life. Cureus.

[22] Hung M, Birmingham WC, Tucker M, Schwartz C, Mohajeri A (2025). Integrating dentistry into interprofessional healthcare: a scoping review on advancing collaborative practice and patient outcomes. Healthcare (Basel).

[23] Patrick DL, Lee RS, Nucci M, Grembowski D, Jolles CZ, Milgrom P (2006). Reducing oral health disparities: a focus on social and cultural determinants. BMC Oral Health.

